# When Equity is Central to Research: Implications for Researchers and Consumers in the Research Team

**DOI:** 10.5334/ijic.2512

**Published:** 2016-12-08

**Authors:** Nicolette Sheridan, Timothy Kenealy, Lynette Stewart, Debra Lampshire, Te Tuhi Robust, John Parsons, Ann McKillop, Yves Couturier, Jean-Louis Denis, Martin Connolly

**Affiliations:** 1 University of Auckland, NZ; 2 Ki A Ora Ngatiwai, NZ; 3 Te Whare Wananga o Awanuiarangi, NZ; 4 University of Sherbrooke, CA; 5 University of Montreal, CA

**Keywords:** equity, health research, consumers, indigenous, engaged scholarship

## Abstract

This paper is a response to our recognition that approaches to equity and consumer involvement in research differed in emphasis between our researchers and jurisdictions. Whilst we shared common aspirations we varied in our priorities between equity groups and methods to represent consumer interests. New Zealand has a historical focus on equity for indigenous Maori and shares with Canada concern about enduring inequalities that affect people’s lives.

## Introduction

In 2011 the New Zealand Health Research Council (HRC) and the Canadian Institutes of Health Research (CIHR) issued a request for proposals for research that focused on older adults with complex chronic conditions. The request for proposals repeatedly called for attention to equity, which is a key indicator of quality of integrated care [1] and can shape a population based strategy for integrated care [2]. Equity is mentioned 12 times in the HRC request for proposal and even more often in the larger Canadian version. Our accepted proposal and subsequent study design necessarily included equity as a foundational component. In this paper we argue that the equity component has direct implications for the composition of the research team and its engagement with consumers (current and past health service users). We also argue that by accepting a role in the iCOACH project all team members implicitly accepted a responsibility to conduct the research in a way that contributes to equity in health outcomes. We describe the fundamental principles behind these implications, how they can be addressed, and provide examples from the iCOACH project. The principles imply that consumers are an integral part of the research process so their selection and roles are subject to the same purpose as others in the research team including contributing to the overall equity aims of the research project.

## Background arguments

The purpose of health services research is to pursue knowledge that will inform and influence health policy, practice and service innovation [3]. At its best this is what Van de Ven has described as engaged scholarship, which is “a participative form of research for obtaining the different perspective of key stakeholder (researchers, users, clients, sponsors, and practitioners) in studying complex problems. By involving others and leveraging the different kinds of knowledge, engaged scholarship can produce knowledge that is more penetrating and insightful than when scholars or practitioners work on the problems alone” [4, p. 9]. The central “problem” considered here is that of health inequities, defined by Whitehead as differences in health that are unnecessary, avoidable, unfair and unjust [5].

Health system restructuring (including in Canada and New Zealand) has emphasised a shift from medical care to primary health care in an attempt to reduce social exclusion and inequities of outcomes in health [6,7]. Health systems are a recognised determinant of health and are thus central to peoples’ rights to health. Politicians, as elected consumer representatives, are accountable for equity in health policy. Health systems that reflect dominant mainstream models of care communicate and enforce norms related to equality (or inequality) in many ways - the provision of entitlements and services, patient and provider treatment within the system, the way services are financed, the extent of social solidarity in resource allocation for health, the degree of transparency and accessibility of relevant information, and the way priorities are set at macro, meso and micro levels of decision-making.

Legitimate claims to entitlements to services must be understood as fundamental rights. The ways in which some people and populations persistently face deprivations across different spheres of life represent not only inherent vulnerability but active processes of exclusion and marginalisation, for which there should be accountability and redress.

Human rights law has been concerned with identifying and protecting people who are consistently disadvantaged, and with social constructs such as gender, race, and class that have maintained this order. Sen argues that a rights-based approach should judge inequality by capability or level of function [8]. Even given equal incomes, a person with a disability does not enjoy the same capabilities as a person with no disability “because he or she suffers from a “conversion handicap”, a differential ability to convert resources into actual opportunities to enjoy good living and to effectively enjoy rights” [8, p. 258]. “Conversion” capabilities are influenced by individual states of ill-health and resources, but they are also heavily influenced by the nature of society and legal frameworks. Poverty has been recognised as more than a lack of money; it can be a result of, and reinforced by, discrimination. Relative differences in income can translate into absolute differences in capabilities. Invariably women, racial and ethnic minorities, older people (particularly the very old), people with disabilities and other marginalised populations, are disproportionately represented among the most economically disadvantaged and whose effective enjoyment of rights is most impaired.

In the context of research on ethnic minorities, Smith states “The word itself, ‘research’ is probably one of the dirtiest words in the indigenous world’s vocabulary” [9, p. 1]. As far back as the eighteenth century “competitive collecting” of territories, new species of flora and fauna, mineral resources and of cultures reaffirmed the Wests’ view of itself as the arbitrator of what counts as legitimate knowledge. Unless research teams value their common purpose of challenging inequities they can too easily perpetuate disadvantage for the same populations.

Smith further argues that because of a history of mistrust and misuse of indigenous knowledge, it can be highly problematic for academics who move across the boundaries of indigenous and urban, institution and community, politics and scholarship to be taken seriously within the academy [9]. A research team engaged in such difficult work needs leadership, determination and a dedication to longer term equity outcomes. Freire referred to this as praxis [10], with communication and mutual support necessary for effective action. This is in contrast to passive aggressive resistance where individuals and teams support equity, for example, in words but not in deeds [11].

Academics commonly invest in discipline-specific knowledge and academic freedom is underpinned by the notion of independence. Both can perpetuate an insularity of discipline that distances researchers in one discipline from those in other disciplines. This is challenging for any team that also seeks to work across divides of discipline, jurisdiction, ethnicity or culture and institutional hierarchy. These challenges create a potential to distance and marginalise consumer participants, which needs to be actively mitigated and managed within the team.

## Equity in the RFP and grant proposal documents

In the Canadian request for proposal, equity was cited with respect to access to, and experience of, health services by “vulnerable populations”. These groups were variously listed in relation to income, immigrant status, chronic conditions, age, socio-economic status, gender/sex or sexuality, developmental or functional disability, inability to communicate effectively, race/ethnicity (including First Nations, Inuit, Aboriginal peoples and other undefined cultural groups) and geography (rural, remote and circumpolar). There was no priority accorded between these equity groups; all were important. The research should address improving health services for those “who are at greater risk of poorer health outcomes and experiencing challenges in equity of access to [community based primary health care], and by addressing individual, social and structural determinants of health that lead to or reinforce conditions of vulnerability (e.g., stigmatization, migration).” While the New Zealand request for proposal offered a similarly inclusive definition of vulnerable populations, it added that the definition “encompasses the populations of priority to the HRC – Māori, Pacific Peoples, children, youth, older adults, and those with impairment living in a disabling society.” Research should contribute to equity “by focusing on vulnerable subgroups”.

Our funded grant proposals necessarily responded to the funders’ emphasis on equity. The proposal documents from each country were almost identical except for an additional section in the New Zealand document about “Responsiveness to Māori”. Both documents acknowledged each other’s health equity issues, including acknowledgment that, in New Zealand, chronic conditions account for a higher proportion of illness and death among Māori, Pacific peoples and people on low incomes than among the general population [12,13] and that some vulnerable populations such as Māori in New Zealand have much shorter life-expectancies and face conditions associated with older age much earlier in their life-course. Both proposals cited a World Health Organization statement that successful primary health care can contribute to the development of health equity, including “the whole spectrum of unnecessary, avoidable and unfair differences in health” [14, p. 24].

## Engaging with consumers: necessary by principle and methodology

Consumers need to be part of the research team both by right and as part of a methodological and political response to addressing equity issues within the research. Consumers bring cultural balance due to a breadth of experiences, values, priorities and relationships that can disrupt assumed wisdoms [15] of other researchers. Their very presence can sanction participation by, and data collection from, other consumers. The consumers who add value to a team include those from populations that the research team members are not themselves part of, especially the target populations addressed by the project. Nelson et al. advise teams to seek consumers who are able to see and articulate issues beyond their own personal experience, are concerned about more than one issue or agenda, and have recognised status as representative of a community served by the part of the health system under investigation [16].

Well-established ways to engage with consumers include seeking input and advice from community leaders, who may further identify opinion leaders within their community. Leaders of special interest consumer groups (often specific disease-focussed or locality-focussed) and leaders of health and social service non-governmental organisations are other obvious and valuable sources of consumer and community input. A relatively new and increasing role is that of the academic consumer, who may have formal status representing a health consumer group, and who is an academic in their own right.

The aims of a project are further supported when academic researchers are themselves consumers (patients or carers) and/or members of equity groups relevant to the project. Clinicians, in particular, will often have insights due to the privileged relationships that are a consequence of their professional roles.

## Implications and examples from iCOACH

The principles and responsibilities for supporting equity apply equally to all iCOACH team members who comprise academic, clinical and indigenous researchers at different stages of career, doctoral students, academic consumer researchers, consumers, and government policy advisors. The following three examples show efforts to establish important relationships and processes so that the research will have greater value to those consumers - individuals, families, and populations - who experience the greatest inequity in health outcomes. In addition, Table 1 offers examples of activities to address equity at each stage of the research process.

**Table 1:** Examples of activities to address equity in the research process.

Project stage	Activities to address equity

RFP	Funder defines research scope to include equity
Building team	Include academic researchers with equity, indigenous and consumer knowledge and experience
Partners	Include partners with equity, indigenous and consumer knowledge and experience; partners may have their own processes of consumer engagement
Grant writing and associated decision-making	Equity outcomes are reflected in high-level aims of project; equity, indigenous and consumer literature and data is represented; research methods are appropriate to data collection from equity, indigenous and consumer groups; formal consultation with, and approval from, head of indigenous research as condition of institutional approval; formal consultation with equity groups required by funding body; letters of support from partners reflect engagement
Ethics application and associated decision-making	Address Māori obligatory (NZ), address other equity groups if identified; consultation with equity groups and indigenous as a requirement for ethics approval
Instrument selection and development	Include instruments that enquire about equity dimensions, include established measures
Case selection	Select cases/participants that include minority populations and known equity issues
Data collection Transcribing	Conduct interviews in a culturally appropriate manner Transcribing and translation, for example by person of the same language and cultural background as the participant
Data analysis	Data analysis is undertaken with consumers and partners
Governance and project management	Formal terms of reference with partners that support an equity agenda; governance that includes consumer and indigenous voice and end-user voice
Findings	Prioritise and present findings that highlight inequity and actions to promote equity
Dissemination	Partners and consumers participate in dissemination of findings to local communities. Researcher advocate for equity with people who can influence policy and practice

### Example 1: Terms of Reference

In a cross-cultural context we sought to make the research relevant to a case study organisation, which represented the interests of indigenous patients and their families. The research team were formally welcomed in a pōwhiri (traditional ceremony) before the research began. Terms of Reference framed the relationship between the case study organisation and iCOACH researchers setting out the purpose, methods for working together, and contributions including costs. Purpose emphasised the organisation’s philosophy and delivery of Whānau Ora (family health), a New Zealand interagency approach implemented through community-based primary health care. Methods were shared, discussed and modified; engagement occurs at all stages of the research, including the dissemination of findings. The agreement specifies that either party can veto publication of specific points or conclusions, but will not unreasonably withhold approval. This provision will outlast the five year term of the project and reflect the spirit of this agreement. At the conclusion of the study the research team will initiate a poroporoaki (farewell ceremony).

### Example 2: Questionnaire development

Sixteen consumers who met the inclusion criteria around age, gender, ethnicity, geography and condition as patient and carer participants contributed to the development of the patient and family carer questionnaires. This included the types, language, structure and order of questions. Validated research instruments, such as “Hua Oranga” [17] were also included in the overall patient and carer questionnaires after consultation with academic consumer researchers and consumers, and were reassessed when piloting. Hua Oranga, a measure of Maori mental health outcome, assesses effectiveness of treatment and care and uses data from three groups - patients, providers and carers. It is underpinned by the theoretical constructs of “Te Whare Tapu Wha” a well-established Māori health model [18] that aligns with health promotion values. While the worth of this instrument was debated by some researchers, many of the consumers, particularly those from minority groups, immediately understood and valued the questions, and supported its inclusion. The researchers agreed to include Hua Oranga, which led to important insights that would not otherwise have been uncovered. Questionnaires developed within the iCOACH project were constrained by the terms of the RFP and the contracted requirements of the grant proposal.

### Example 3: Academic consumer and academic indigenous partners

We adopted a strategy of partnership where we/the iCOACH team could learn and be guided by academic consumer and academic indigenous partners. These roles were pivotal throughout the project and supported practical ways of engaging with specific consumers and communities. One consumer researcher is an experience-based expert who uses health services and lives with a chronic condition. This person has a formal role in the academy and has multiple current and previous roles advocating for consumers in government advisory roles and with international organisations. A second consumer advocate has held national academic governance roles and regional and local health service governance roles, as well as indigenous policy and service governance and advisory roles. A third consumer advocate is also a senior indigenous academic with expertise in education. This person has held leadership and governance roles in NGOs that provide health and social services to a large indigenous community.

## Conclusion

As a research team we accepted a mandate to conduct a research project that included equity as a core principle. We believe that engaging consumers in research should be seen in the wider context of engaging with people and methods that would serve the purpose of advancing equity of knowledge and outcomes for the population which is the focus of this project. The grant proposal identified a range of equity groups but ethnicity has been the most consistent focus, particularly for the New Zealand team. While we have argued that equity is an important area to address we have also indicated why this can be difficult and that some past research has either ignored or even harmed those who would be most affected by it, especially indigenous peoples.

We sought scholarship, connectivity and diffusion of insights as central elements of the research teams’ engagement strategy. We identified ways in which equity for consumers and especially indigenous peoples can be addressed, and offered examples from the iCOACH project. We have not yet met the ideals we set out above. We offer our reflections in the hope of guiding other researchers, and to challenge ourselves to do better in an area that has historically been poorly addressed.
